# *Case Report: De novo KLHL24* Gene Pathogenic Variants in Chinese Twin Boys With Epidermolysis Bullosa Simplex

**DOI:** 10.3389/fgene.2021.729628

**Published:** 2021-11-05

**Authors:** Xiaojing Xu, Juan Zhao, Chao Wang, Xiaoxuan Qu, Menglong Ran, Fang Ye, Ming Shen, Kundi Wang, Qi Zhang

**Affiliations:** ^1^ Department of Pediatrics, China-Japan Friendship Hospital, Beijing, China; ^2^ Department of Dermatology and Venereology, Peking University First Hospital, Beijing, China; ^3^ Beijing Key Laboratory of Molecular Diagnosis on Dermatoses, Beijing, China

**Keywords:** KLHL24, *de novo* pathogenic variants, epidermolysis bullosa, skin defect, follow-up

## Abstract

**Objectives:** The aim of this study was to determine the molecular etiology and clinical manifestations of a pair of Chinese twins affected with epidermolysis bullosa simplex. Pediatricians should pay attention to the early genetic diagnosis of this disease.

**Methods:** Histopathological examination of HE-stained skin, electron microscopy of biopsied normal skin, and whole-exome sequencing was performed to assess pathogenicity and conservation of detected mutations. Two years later, the cutaneous and extracutaneous manifestations of the twins were comprehensively evaluated.

**Results:** *A de novo* pathogenic variant c.2T>C (p.M1T) in *KLHL24* (NM_017,644) was identified in both twins. The characteristics of extensive skin defects on the extremities at birth and the tendency to lesson with increasing age were confirmed. No positive sensitive markers, such as B-type natriuretic peptide, cardiac troponin I, for cardiac dysfunction were detected.

**Conclusions:** The *de novo* pathogenic variants c.2T>C (p.M1T) in *KLHL24* (NM_017,644) contributes to the development of epidermolysis bullosa. Genetic diagnosis at birth or early infancy can better predict the disease prognosis and guide the treatment.

## Introduction

Inherited epidermolysis bullosa (EB) is a genetically heterogeneous disorder, characterized by skin fragility annexed with the formation of blisters and skin erosion in response to minor mechanical trauma.1 Currently, over twenty genes that encode structural proteins within keratin intermediate filaments, focal adhesion desmosome cell junctions, and hemidesmosome attachment complexes have been reported in the pathogenesis of EB. (Has and Fischer, 2018; Vahidnezhad et al., 2018). Clinically, EB is classified into four major groups based on the plane of cleavage within the skin, *viz*. epidermolysis bullosa simplex (EBS), junctional epidermolysis bullosa (JEB), dystrophic epidermolysis bullosa (DEB), and Kindler epidermolysis bullosa (KEB) (Bardhan et al., 2020). The diagnosis and classification of EB are based on the morphological analysis of a skin sample using immunohistologic methods and on the analysis of the pathogenic variants of the candidate genes. (Has and He, 2016; Has and Fischer, 2018). As the most common type of EB, EBS is mainly caused by monoallelic pathogenic variants in *KRT5* (MIM: 148,040) and *KRT14* (MIM: 148,066), which encode keratin 5 and keratin 14, respectively. In addition, some cases of EBS were reported to associate with other pathogenic variants in *PLEC* (plectin), *EXPH5* (exophilin-5), *DST* (dystonin, 230-kDa bullous pemphigoid antigen), and *CD151* (member of the tetraspanin superfamily). (Karamatic Crew et al., 2004; Groves et al., 2010; McGrath et al., 2012; Bolling et al., 2014; Bardhan et al., 2020). Recently, pathogenic variants in *KLHL24* (MIM: 611,295) encoding the Kelch-like protein 24 have been identified in cases with skin fragility and progressive thickening of the nails by whole-exome sequencing. To date, about 40 individuals with monoallelic pathogenic variants of *KLHL24* have been reported. (He et al., 2016; Lin et al., 2016; Lee et al., 2017; Alkhalifah et al., 2018; Yenamandra et al., 2018; Grilletta, 2019; Hachem et al., 2019; Schwieger-Briel et al., 2019). *KLHL24* is part of the family of more than 40 genes with a Kelch-like motif, and it partially forms the ubiquitin-ligase complex. (Dhanoa et al., 2013). These pathogenic variants caused the loss of the first 28 amino acids of the encoded protein. The mutant protein promotes excessive ubiquitination and degradation of KRT14. The above observations have invoked a new mechanism that is germane to inherited skin blistering, namely, dysregulation of autoubiquitination. (He et al., 2016). Most *KLHL24* positive patients carry a heterozygous pathogenic variant in the first codon that affects translation initiation. (He et al., 2016; Lin et al., 2016; Lee et al., 2017; Alkhalifah et al., 2018; Yenamandra et al., 2018).

The clinical diagnosis for EB can be difficult at birth or in the early infancy, even for experienced dermatologists, particularly without an established family history of the disease. An important part of EB research lies in the diagnosis and classification of the disease at the early stage. The optimal treatment regime for disease complications has to be assessed for a long time. Here, we reported a case of twin boys with *de novo KLHL24* pathogenic variants. This is the first study to describe the pathogenic variant in *KLHL24* affecting Chinese twins. The twin brothers were diagnosed, screened, and treated effectively at the early stage by pediatricians. Meanwhile, cutaneous and extracutaneous manifestations were evaluated at the age of two. This case report will help pediatricians, not confined to dermatologists, to pay enough attention to the early diagnosis and long-term management of EB.

## Case Report

**FIGURE 1:**
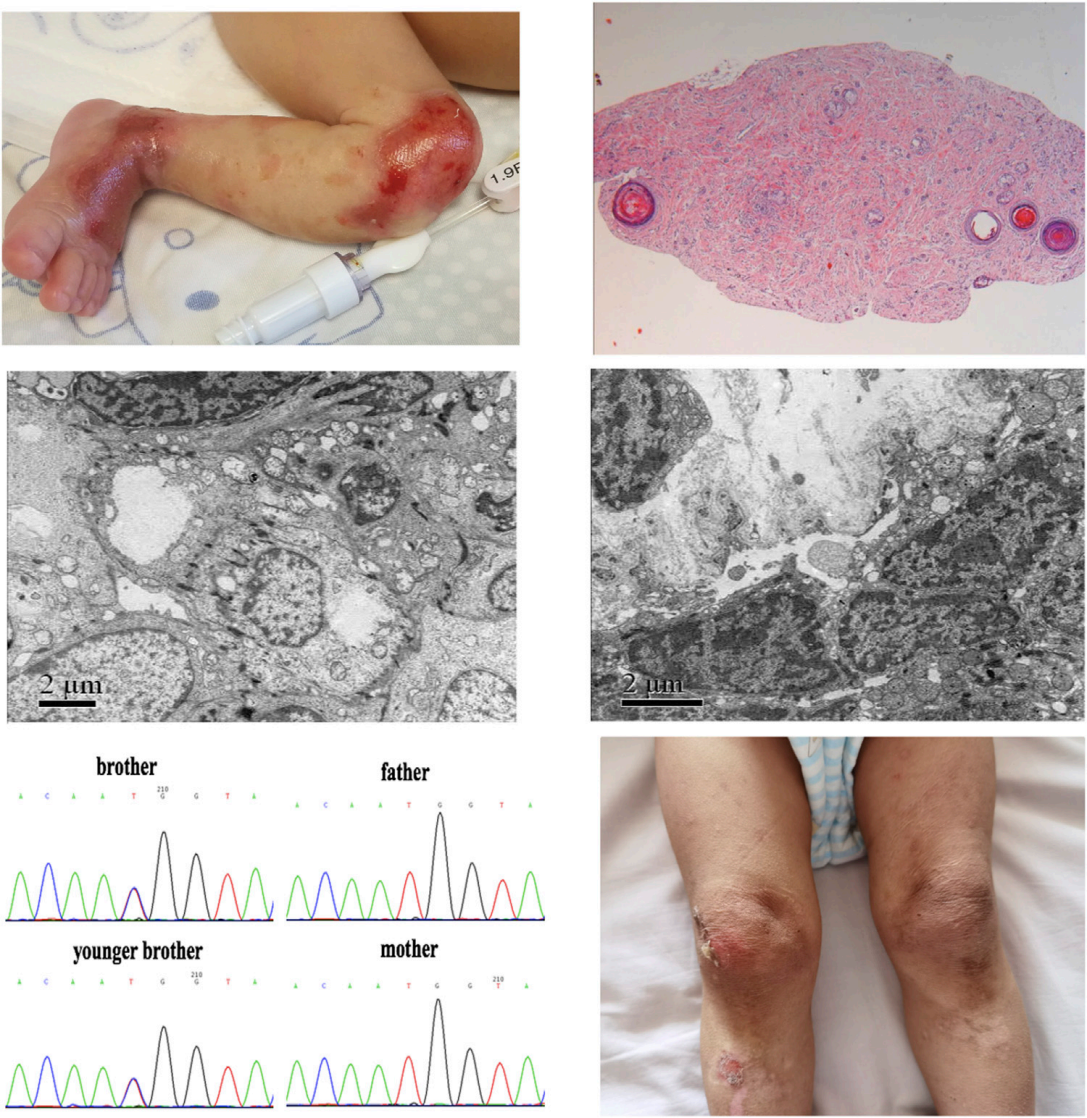
Skin image, pathology, and *de novo* pathogenic variants of *KLHL24* in the twin brothers. **(A)**Picture of the older brother’s left leg at birth demonstrated congenital skin defects. **(B)**Histopathological examination of HE-stained skin in the older brother **(C)** Skin performance under EM in the older brother **(D)** Skin performance under EM in the younger brother **(E)** *De novo* pathogenic variants of c.2T>C (p.M1T) in *KLHL24* of this ancestry **(F)** Picture of younger brother’s legs at the age of 2 years demonstrated skin damage, pigmentation and scar.

The dichorionic twin boys in this report were born at the 32nd week of gestation as a result of a large intracranial hemorrhage in their mother, who had a history of multiple spontaneous abortions under diverse complications, including antiphospholipid antibody syndrome and subclinical hypothyroidism during pregnancy, and she took multiple medications during pregnancy, including methylprednisolone, hydroxychloroquine, and aspirin. The older brother’s body weight was 1,380 g, less than third percentile of typical boys of the same gestational age. At birth, he presented with extensive areas of denuded skin involving the limbs, knees, wrist joints, and ankle joints (Figure 1A). The younger brother’s body weight was 1,650 g, which ranges between the 25th and 50th percentiles for boys of the same gestational age. His skin had the same appearance as his brother’s. New skin defects occurred on the twins faces after positive-pressure ventilation. At birth, both the white blood cell count and neutrophil count of the two brothers were low. The white blood cell count of the elder brother was 1.94*10^9^/L and the neutrophil count was 0.2*10^9^/L, and that of the younger brother was 1.58*10^9^/L and 0.29*10^9^/L respectively. The course was complicated by *Serratia marcescens* sepsis as a result of preterm labor. The twins homocysteine levels at birth were 4.42 and 4.61 μmol/L (normal ≤15 μmol/L) respectively. The results of echocardiography indicated congenital heart disease and atrial septal defect (secondary foramen type), and the degree of interruption was 6 and 5 mm, respectively. The remaining systemic examination was normal. Histopathological examination of hematoxylin-eosin (HE)–stained skin and electron microscopy (EM) of normal skin biopsy were performed in a reference center for EB. In the older brother, pathology showed no *epidermis* or intradermal vascular hyperplasia (Figure 1B). EM revealed cleavage within the epidermal basal layer, some epidermal cells with a large amount of melanin deposition, reduction in the local density of the superficial dermis, and partial basal cell degeneration with vacuolar changes (Figure 1C). In his younger brother, histopathological examination showed no *epidermis* and dermis with only a lipid membrane structure. EM also revealed cleavage within the epidermal basal layer and the substrate, incomplete basal cells within the dermis, and reduction in the local density of the superficial dermis (Figure 1D). These results collectively suggest EBS. Soon thereafter, a whole-exome sequencing analysis was performed of peripheral blood DNA for this family. We identified the *de novo* variants c.2T>C (p.M1T) in *KLHL24* (NM_017,644) from the two boys, which were previously reported to be pathogenic. (He et al., 2016). We have applied ACMG, PolyPhen-2 and PROVEN criteria to prove the pathogenicity of this mutation c.2T>C. Moreover, variant c.2T>C was absent in cohorts of healthy control subjects in dbSNP, 1,000 Genomes, the Exome Variant Server, and the ExAC Browser in previous report (He et al., 2016). Sanger sequencing confirmed these *de novo* pathogenic variants (Figure 1E). Treatment consisted largely of supportive care, including wound care, as well as prevention and treatment of complications. Mupirocin ointment and recombinant bovine basic fibroblast growth factor were mixed at a 1:1 ratio, then above medicine was applied on the oil gauze, and finally the oil gauze was covered the skins wound. The treatment was carried out every other day on the twin boys. After about 1 month, the stability of the skins was enhanced gradually, above skin treatments were carried on when necessary.

At the age of 2 years, we followed up with the two brothers. There were old scars, pigmentation, nail thickening, and yellowing, no joint contracture and functional damage, and few new blisters (Figure 1F). Their head circumferences were below the third percentile of boys after adjusted age, and their heights and body weights were between the third and 10th percentiles. Considering the existence of extrauterine growth retardation, it may be related to the lack of functional training, regular follow-up, and the late addition of complementary food. Both brothers were assessed for Gesell Developmental Observation, and they showed mild retardation in adaptability, fine movement and personal social interaction, moderate retardation in language, and normal serum myocardial enzymes (except the MB isoenzyme of creatine kinase is higher than normal in elder brother). The elder brother’s brain MRI showed myelination delay, and echocardiography showed atrial septal defect (5 mm) and a small amount of tricuspid regurgitation, yet the results of the younger brother’s brain MRI and echocardiography were normal.

## Discussion

*KLHL24* was first reported by He et al., (He et al., 2016), who discovered heterozygous pathogenic variants of this gene in 5 unrelated individuals with EBS. (Lin et al., 2016). *KLHL24* is expressed in multiple tissues, including heart, brain, liver, skeleton muscle, kidney, pancreas, placenta, lung, and peripheral blood, as well as in the main skin cell types: keratinocytes, fibroblasts, and melanocytes. (He et al., 2016; Lin et al., 2016). It was of interest to note that all 26 previously reported patients harbored monoallelic pathogenic variants in the *KLHL24* translation start codon, c.1A > G, c.1A > T, c.2T > C, c.3G > T, c.3G > A, with a high rate of *de novo* and recurrent pathogenic variants. (Has and Fischer, 2018)^,^ (Has, 2017) In our case, it is the first to describe c.2T>C pathogenic variant in *KLHL24* affecting twins in China and it was correlated with EB simplex. He et al. (He et al., 2016) also found truncated *KLHL24* resulting from the start codon mutations and the use of a downstream methionine initiation codon. Abnormal intermediate filaments in keratinocytes and fibroblasts, with evidence for irregular and fragmented KRT14, and data to support an altered balance in the stability and degradation of this keratin (He et al., 2016).

The clinical manifestations of our cases included extensive skin defects on the extremities at birth, leaving hypopigmentation and atrophy with a whirled pattern, in accordance with the characteristic of *KLHL24*. In addition, early blistering occurred often in the trunk and upper limbs, especially after the compression or friction. These lesions were typically healed quickly with subtle atrophic scarring. Based on the results of other studies, blistering persists throughout childhood but tends to lesson with increasing age. (Hachem et al., 2019). Nail defects and oral ulceration are common, whereas transient milia also occur. Dyspigmentation is not a prominent feature. Other reported features include cutaneous findings, such as loss of dermatoglyphics, hypohidrosis, and congenital malrotation of the great toenails, besides mental problems. (Yenamandra et al., 2018). Whether these symptoms are related to *KLHL24* requires further investigations. After 2 years of follow-up in our study, we found that the skin defects became milder, nails became thicken and yellowing, the oral ulceration was not obvious.

Indeed, the wide tissue distributions of *KLHL24* suggest that pathogenic variants could affect organs other than the skin. Schwieger et al. (Schwieger-Briel et al., 2019) found evidence of dilated cardiomyopathy in 8 of 20 EBS-*KLHL24* patients (40%), with the youngest being 25 years. He et al. (He et al., 2016) also noticed dilated cardiomyopathy in a 43 year-old patient, although the age of onset in this patient was unclear. In our report, the cardiac ultrasound examinations of the boys indicated congenital heart disease (atrial septal defect). Other sensitive markers, such as B-type natriuretic peptide, cardiac troponin I, for cardiac dysfunction were proved negative until hospital discharge (except for the MB isoenzyme of creatine kinase is higher than normal in the elder brother). Because of the skin damage and other problems caused by the disease, the children need special care in daily life which produced heavy financial burden for the family. At 2 years of birth, they were comprehensively evaluated by pediatricians, dermatologists, and neurologists. The dichorionic twin boys in this report were born at the 32nd week of gestation. At the 2-year follow-up, except for old scars, we only found the elder brother’s brain MRI showed delayed myelination, and echocardiography showed atrial septal defect (5 mm) and a small amount of tricuspid regurgitation. Their phenotypes were compared with previous cases reported with c.2T>C variant in *KLHL24* (Table 1). As myelination is a progression phenotype, later examinations will be required to confirm their brain developmental status. Congenital heart disease is very common in premature infants. Clinicians also need further follow-ups to determine the future treatment plan. Future cardiac complications may emerge with age, and this should be a focus of the treating physician. Considerably different phenotypes of pathogenic variants have been reported within EB subtypes. There were correlations between phenotypes and genotypes in EB. *KLHL24* pathogenic variants were associated with the mild phenotype, such as EB simplex.

**TABLE 1:** Summary of clinical features of individuals with c.2T>C pathogenic variant in *KLHL24*.

Case	Age (Y)	Sex	Areas of birth damage	Scarring at sites of birth damage	Milia	Nail	Oral	Hair	Cardiac symptoms/Clinical features	CK in U/L (normal value)	CKMB in ng/ml (normal value)	Pro-BNP or BNP in pg/ml (normal value)	Development
1 Lee et al. (2017)	9	M	Legs, wrists	+	+	+	−	−	NA	NA	NA	NA	NA
2 He et al. (2016), Schwieger-Briel et al. (2019)	4	M	Legs, arms, wrists	+	+	+	−	−	Tachycardia, extrasystoles at 6Y	182 (<168)	7.7 (<6)	82 (<390)	Delay of cognitive and motor development
3 Hachem et al. (2019)	7	M	Legs, arms, wrists, buttocks, left mammary region	+	+	+	+	+	Normal	Normal	Normal	Normal	NA
4 Hachem et al. (2019)	5	M	Legs, arms, abdomen, wrists	+	+	+	−	−	Normal	Normal	Normal	Normal	NA
5 (Twin 1, elder brother)	2	M	Legs, arms, wrists	+	+	+	−	−	Atrial septal defect (5 mm) and small amount of tricuspid regurgitation	124 (<200)	4.3 (<4.0)	47 (<100)	Delayed myelination, mild retardation in adaptability moderate retardation in language
6 (Twin 2, younger brother)	2	M	Legs, arms, wrists	+	+	+	−	−	Normal	103 (<200)	2.74 (<4.0)	22.6 (<100)	Mild retardation in adaptability, moderate retardation in language

NA: not applicable

Although there is currently no effective treatment for EB, genetic diagnosis at an early age can better predict the prognosis and guide the treatment. We here recommend both genetic and prenatal diagnoses to reduce the incidence of this disease and improve the quality of life.

This study was supported by the Medical and health science and technology innovation project of the Chinese Academy of Medical Sciences (2018-12M-1–003), and approved by the Ethical Committee, China-Japan Friendship Hospital. All study protocols and the report of the clinical research findings are in accordance with federal laws and institutional regulations in China and approved by the institutional review board. Written informed consent was obtained from the father (legal guardian) of the twins to agree to the participation and reporting of the clinical records, as well as the publication of this case report and all information and any accompanying images from the family.

## Data Availability

The datasets for this article are not publicly available due to concerns regarding participant/patient anonymity. Requests to access the datasets should be directed to the corresponding author.
