# Supplementary figures and images for: Case Report: De novo KLHL24 Gene Pathogenic Variants in Chinese Twin Boys With Epidermolysis Bullosa Simplex
Source: Front Genet. 2021 Nov 5;12:729628. doi: 10.3389/fgene.2021.729628 (PMC8602111; doi:10.3389/fgene.2021.729628)

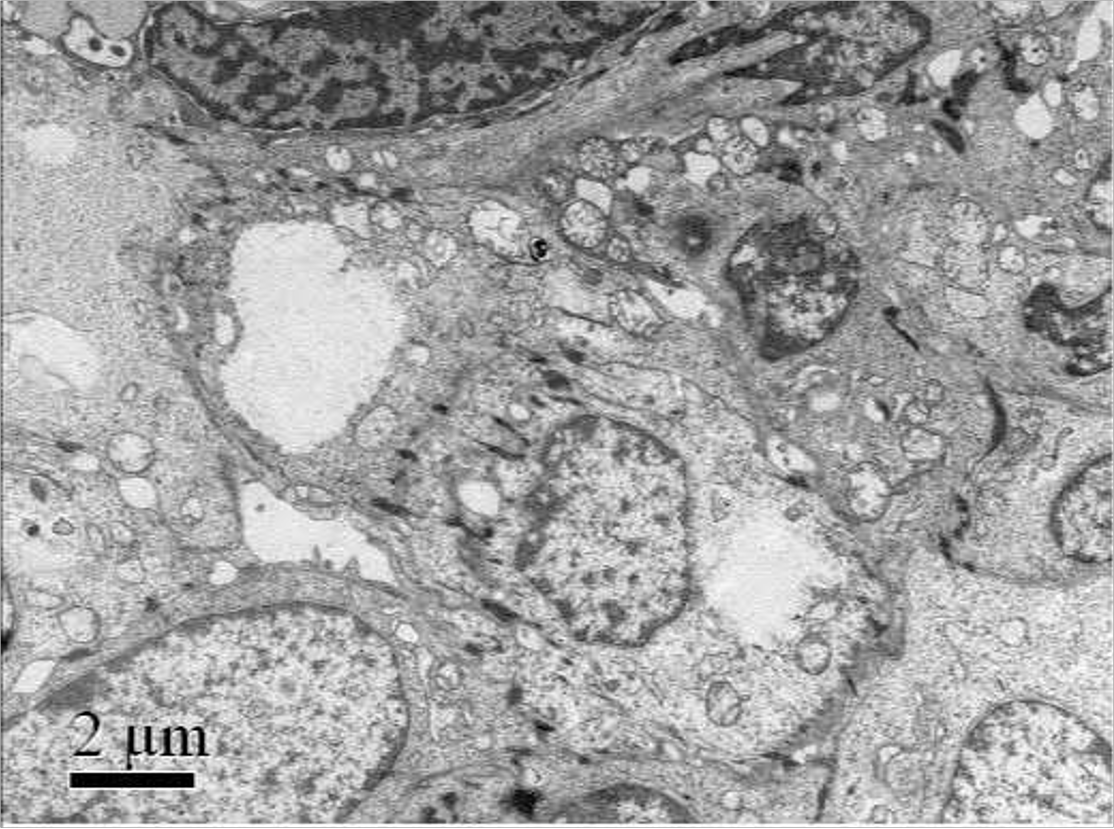

Supplement: Supplementary file 1 [file Image3.TIFF]

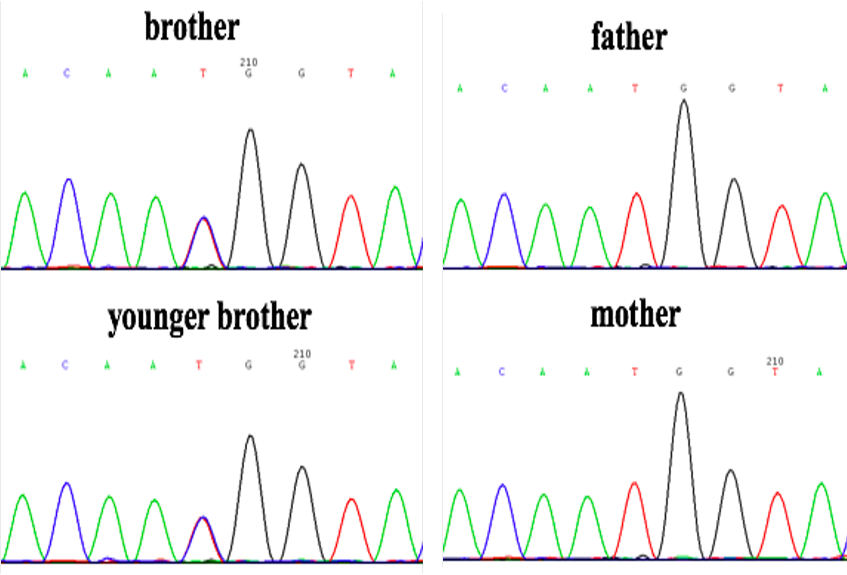

Supplement: Supplementary file 2 [file Image1.TIFF]

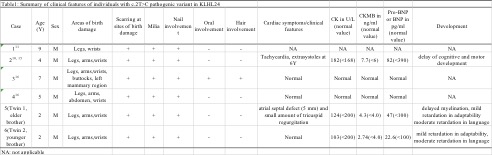

Supplement: Supplementary file 3 [file Image5.JPEG]

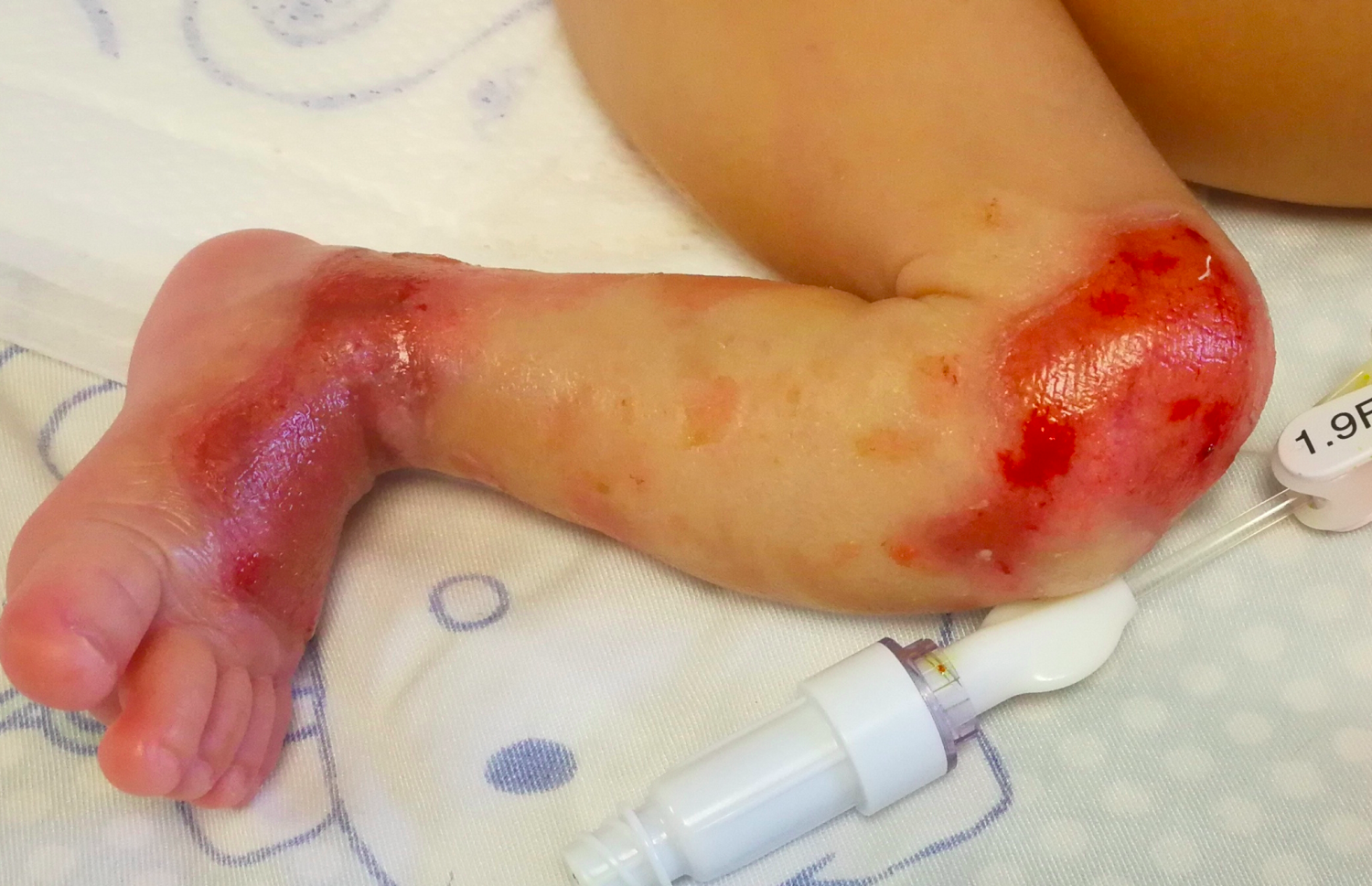

Supplement: Supplementary file 4 [file Image6.TIFF]

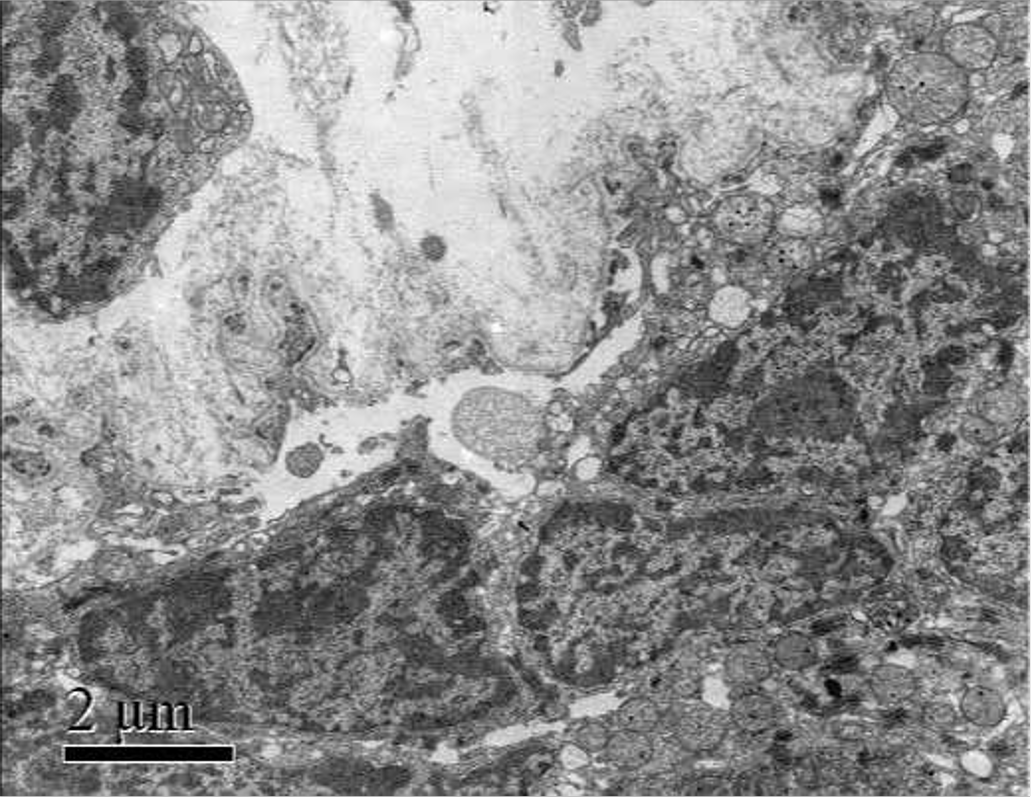

Supplement: Supplementary file 5 [file Image2.TIFF]

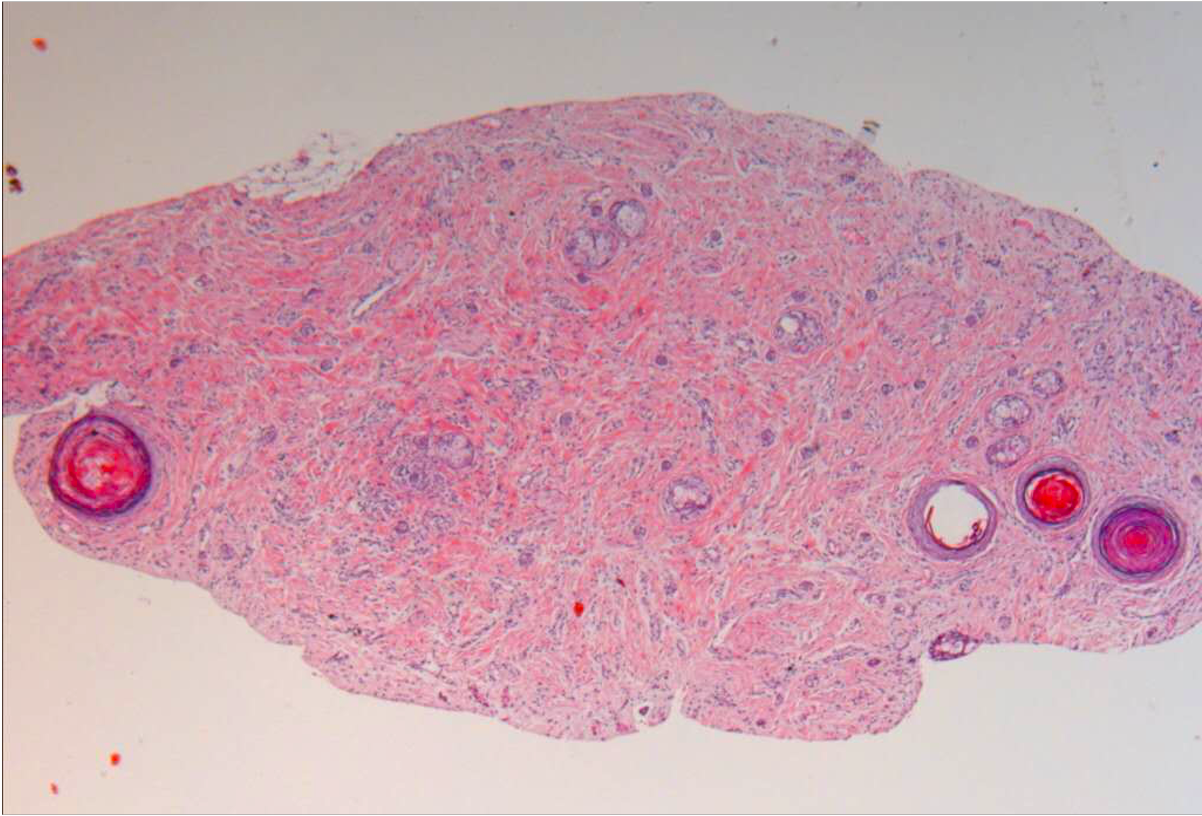

Supplement: Supplementary file 6 [file Image4.TIFF]

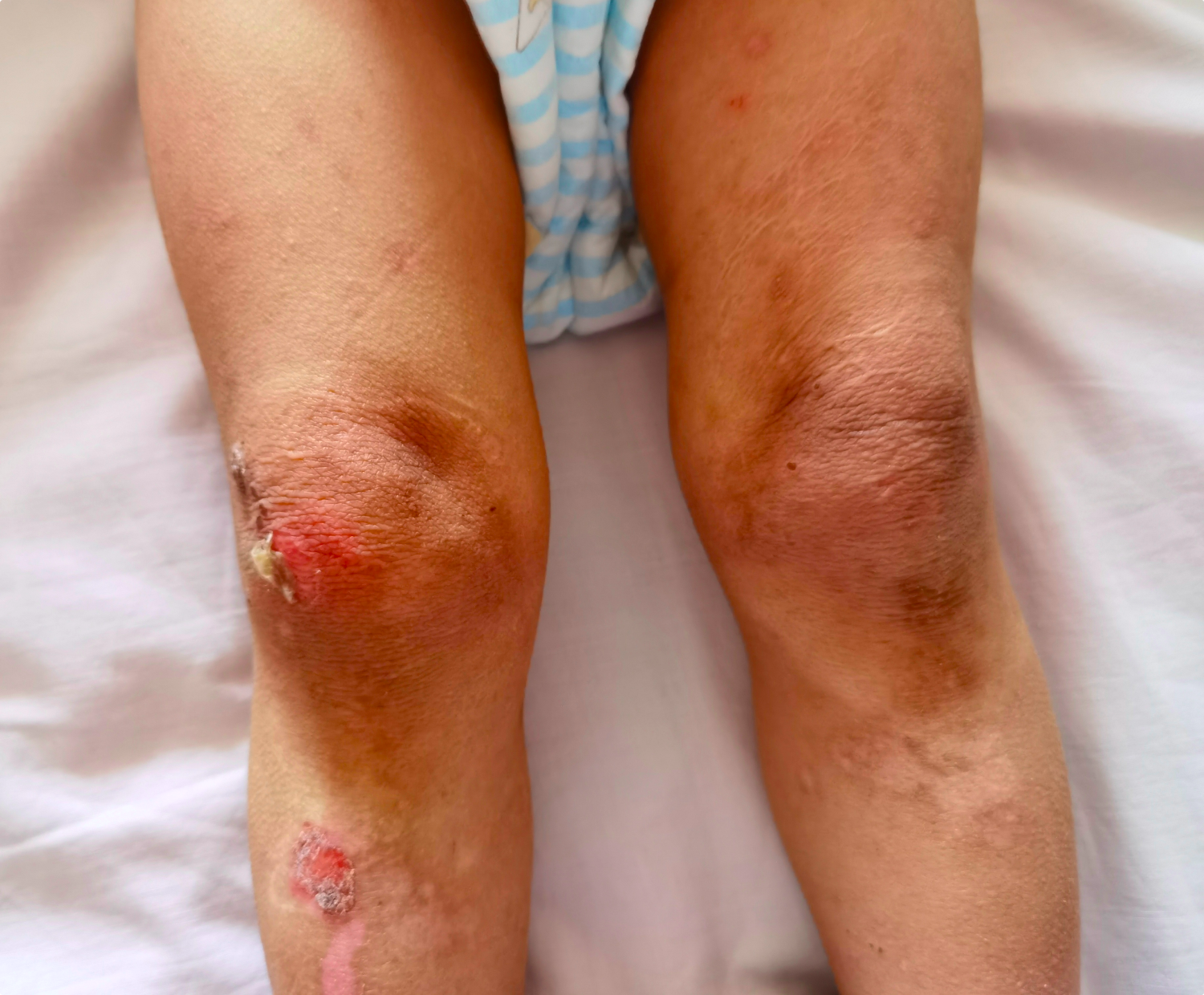

Supplement: Supplementary file 8 [file Image7.TIFF]
