# Supplementary material for: Case Report: De novo KLHL24 Gene Pathogenic Variants in Chinese Twin Boys With Epidermolysis Bullosa Simplex
Source: Front Genet. 2021 Nov 5;12:729628. doi: 10.3389/fgene.2021.729628 (PMC8602111; doi:10.3389/fgene.2021.729628)
Supplement: Supplementary file 7 [file DataSheet1.docx]

**2013 CARE Checklist**

1. **Title** – de novo KLHL24 gene pathogenic variants in Chinese twin boys with epidermolysis bullosa simplex, case report.
2. **Key Words** – KLHL24; de novo pathogenic variants; epidermolysis bullosa; skin defect; follow-up
3. **Abstract** – structured
   - Introduction – What is unique about this case and what does it add to the scientific literature?

In our case, it is the first to describe c.2T>C pathogenic variant in KLHL24 affecting twins in China. Pediatricians should pay attention to early genetic diagnosis of this disease to guide the prognosis and treatment.

- - The patient’s main concerns and important clinical findings.

In the twin boys, the characteristics of extensive skin defects on the extremities at birth and they tends to lesson with increasing age were confirmed.

- - The primary diagnoses, interventions, and outcomes.

The twins were diagnosed as epidermolysis bullosa simplex through histopathological examination of HE stained skin, electron microscope of normal skin biopsy, and whole exome sequencing analyses. The de novo pathogenic variants c.2T>C (p.M1T) in KLHL24 were identified in the twins. Two years later, the twins’ skin defects tended to lesson with increasing age.

- - Conclusion – What are one or more “take-away” lessons from this case report?

Early gene diagnosis technology can better guide epidermolysis bullosa patients’ prognosis and treatment.

1. **Introduction** – Briefly summarizes why this case is unique and may include medical literature references.

We reported the case of twin boys with de novo KLHL24 pathogenic variants and followed up after 2 years. It is the first to describe pathogenic variant in KLHL24 affecting twins in China. We hope that pediatricians, not confined to dermatologists, would pay enough attention to the early diagnosis and long term management of EB through this case report.

1. **Patient Information**
   - De-identified patient specific information.

Yes.

- - Primary concerns and symptoms of the patient.

The patients presented with extensive areas of denuded skin involving the limbs, knees, wrist joints, and ankle joints. New skin defects occurred on the twins in the course of treatment continuously.

- - Medical, family, and psychosocial history including relevant genetic information.

Their mother had a history of multiple spontaneous abortions under diverse complications, including antiphospholipid antibody syndrome and subclinical hypothyroidism during pregnancy, and she took multiple medications during pregnancy, including methylprednisolone, hydroxychloroquine, and aspirin. The skins of their parents were normal, and the c.2T>C (p.M1T) in KLHL24 were de novo pathogenic variants.

- - Relevant past interventions and their outcomes.

None.

1. **Clinical Findings** – Describe significant physical examination (PE) and important clinical findings.

The twin boys in this report were born at 32^th^ week of gestation. At birth, they presented with extensive areas of denuded skin involving the limbs, knees, wrist joints, and ankle joints. At first, both white blood cell count and neutrophil count of the two brothers were low. Histopathological examination of hematoxylin eosin (HE)–stained skin and electron microscopy (EM) of normal skin biopsy findings were performed. Pathology showed no epidermis or intradermal vascular hyperplasia in the older brother. EM revealed cleavage within the epidermal basal layer, some epidermal cells with a large amount of melanin deposition, reduction in the local density of the superficial dermis, and partial basal cell degeneration with vacuolar changes. We identified the de novo pathogenic variants c.2T>C (p.M1T) in KLHL24 (NM_017644) of the two boys.

1. **Timeline** – Historical and current information from this episode of care organized as a timeline (figure or table).

| Time line | At birth | One month | 2 years |
| --- | --- | --- | --- |
| Episode | Wound care;Histopathological examination of HE stained skin, electron microscope of normal skin biopsy, and whole exome sequencing analyses | Wound care tends to lesson; The de novo pathogenic variants c.2T>C (p.M1T) in KLHL24 (NM_017644) was identified in the twins. | The twins were comprehensively evaluated. |

1. **Diagnostic Assessment**
   - Diagnostic methods (PE, laboratory testing, imaging, surveys).

PE, histopathological examination of hematoxylin eosin (HE)–stained skin and electron microscopy (EM) of normal skin biopsy, whole-exome sequencing analysis

- - Diagnostic challenges.

The course was complicated by serratia marcescens sepsis as a result of preterm labor.

- - Diagnosis (including other diagnoses considered).

Epidermolysis bullosa simplex, Serratia marcescens sepsis

- - Prognostic characteristics when applicable.

The skin defects tended to lesson with increasing age in epidermolysis bullosa simplex.

1. **Therapeutic Intervention**
   - Types of therapeutic intervention (pharmacologic, surgical, preventive).

Treatment consisted largely of supportive care, including wound care, as well as prevention and treatment of complications.

- - Administration of therapeutic intervention (dosage, strength, duration).

Mupirocin ointment and recombinant bovine basic fibroblast growth factor were mixed in the same amount, then applied on the oil gauze, and finally covered the skins’ wound. The treatment was carried out every other day on the twin boys for about one month.

- - Changes in therapeutic interventions with explanations.

After the de novo pathogenic variants c.2T>C (p.M1T) in KLHL24 (NM_017644) were identified in the twins, our key point of skin wound is basic nursing.

1. **Follow-up and Outcomes**
   - Clinician- and patient-assessed outcomes if available.

After the findings of histopathological examination of hematoxylin eosin (HE)–stained skin, electron microscopy (EM) of normal skin biopsy and pathogenic variants c.2T>C (p.M1T) in KLHL24 (NM_017644) were identified in the twins, epidermolysis bullosa simplex was diagnosed.

- - Important follow-up diagnostic and other test results.

At the age of 2 years, there were old scars, pigmentation, nail thickening and yellowing, no joint contracture and functional damage, and few new blisters in the two brothers.

- - Intervention adherence and tolerability. (How was this assessed?)

The twins visited the pediatric outpatient department of our hospital every 3-6 months.

- - Adverse and unanticipated events.

None.

1. **Discussion**
   - Strengths and limitations in your approach to this case.

Strengths: It is the first to describe c.2T>C pathogenic variant in KLHL24 affecting twins in China and the patients were followed up for 2 years.

Limitations: We collected only 2 cases and had not very much patients’management experience.

- - Discussion of the relevant medical literature.

Some literature suggested KLHL24 could affect organs other than the skin, especially the cardiac complications. In our cases, the cardiac ultrasound examinations of the boys indicated congenital heart disease (atrial septal defect). However, other sensitive markers, such as B-type natriuretic peptide, cardiac troponin I, and MB isoenzyme of creatine kinase, for cardiac dysfunction were proved negative until hospital discharge. At 2 years of the both, we reevaluated the cardiovascular system and all were normal.

- - The rationale for your conclusions.

After the de novo pathogenic variants c.2T>C (p.M1T) in KLHL24 (NM_017644) were identified in the twins, epidermolysis bullosa simplex was diagnosed. The skin defects tends to lesson with increasing age were confirmed.

- - The primary “take-away” lessons from this case report (without references) in a one paragraph conclusion.

The de novo pathogenic variants c.2T>C (p.M1T) in KLHL24 (NM_017644) were correlated with epidermolysis bullosa simplex and suggested a better prognosis compared with other genotypes. Early gene diagnosis technology can better guide the prognosis and treatment. This is the significance of early gene screening diagnosis.

1. **Patient Perspective** – The patient should share their perspective on the treatment(s) they received.

Because of the skin damage and other problems caused by the disease, the children need special care in daily life, and put an extra financial burden on the family.

1. **Informed Consent** – The patient should give informed consent. (Provide if requested.)

Written informed consent was given by the father of the twins for participation and using clinical records. Written informed consent was obtained from the father for publication of this case report and all information and any accompanying images contained within it.

The CARE checklist (and writing outline) have been translated into multiple languages.
